# The mechanism of plasma exosome miR-15a-5p targeting the CF-modified protein IGF1R to regulate alveolar epithelial autophagy and influence pulmonary interstitial fibrosis

**DOI:** 10.1016/j.ncrna.2025.07.001

**Published:** 2025-07-03

**Authors:** Yina Li, Nan Wang, Jinying Hu, Minlan Luo, Na Zhang, Lili Gao

**Affiliations:** Department of Respiratory Medicine, The First Affliated Hospital of Dalian Medical University, Dalian, 116011, People's Republic of China

**Keywords:** Pulmonary interstitial fibrosis, Exosomes, Autophagy, miRNA, Core fucosylation

## Abstract

**Aims:**

This study investigates how plasma exosomal miRNAs regulate core fucosylation (CF)-modified targets to influence autophagy and fibrosis in idiopathic pulmonary fibrosis (IPF), aiming to identify novel therapeutic strategies targeting dysregulated alveolar epithelial cell (AEC) autophagy.

**Materials and methods:**

Plasma exosomes from IPF patients and healthy controls were isolated via ultracentrifugation, validated by TEM, nanoparticle tracking analysis (NTA), and Western blot (CD9/CD81). Exosomal miRNA profiling employed high-throughput sequencing, with TargetScan/miRanda predicting target genes. A549 and MLE-12 cells assessed exosome uptake (PKH67 labeling) and miRNA-mRNA interactions (dual-luciferase assays). CF modification was analyzed via immunoprecipitation/Western blot. In vivo validation used bleomycin (BLM)-induced fibrosis models in alveolar epithelial-specific FUT8-knockout (CKO) mice.

**Key findings:**

IPF plasma exosomes suppressed autophagy and exacerbated fibrosis in AECs. miR-15a-5p was markedly downregulated in IPF exosomes. Overexpression of miR-15a-5p reversed BLM-induced autophagy inhibition and fibrosis. Mechanistically, miR-15a-5p directly targeted IGF1R, a CF-modified protein. Reduced miR-15a-5p elevated IGF1R expression, activating PI3K/AKT to inhibit autophagy and promote fibrosis.

**Significance:**

This study identifies miR-15a-5p as a critical regulator of CF-modified IGF1R in IPF pathogenesis. Its downregulation drives PI3K/AKT-mediated autophagy suppression, accelerating fibrosis. Restoring miR-15a-5p or targeting IGF1R/PI3K/AKT signaling may offer novel therapeutic avenues for IPF.

## Graphical abstract

**Figure:**
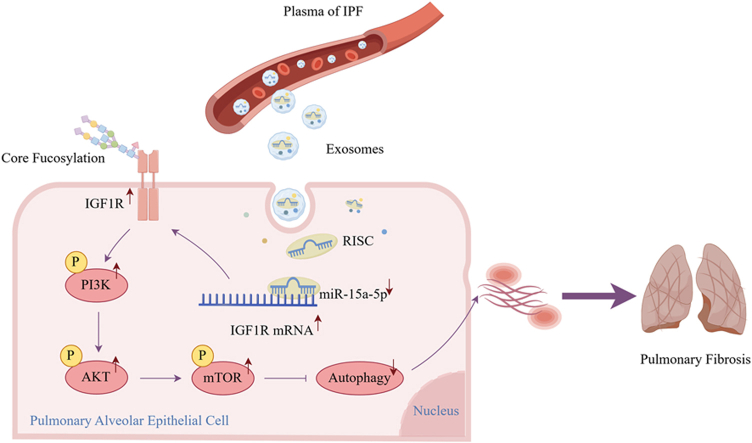

## 1 Introduction

IPF is a chronic, progressive interstitial lung disease of unknown etiology, characterized by poor prognosis, high mortality, and a median survival of 2–4 years post-diagnosis [1,2]. Although pirfenidone and nintedanib are approved therapies that modestly slow disease progression [3,4], no curative treatments exist. Elucidating IPF pathogenesis and identifying novel therapeutic targets remain urgent clinical priorities.

Alveolar epithelial cell (AEC) injury is central to IPF pathogenesis [5]. Damaged AECs initiate aberrant epithelial-fibroblast crosstalk, driving myofibroblast proliferation, excessive extracellular matrix deposition, and tissue remodeling—hallmarks of fibrotic progression [6]. Autophagy, a lysosomal degradation process critical for cellular homeostasis, is paradoxically suppressed in fibrotic lungs [7,8]. Our prior work demonstrated impaired AEC autophagy in IPF [9], suggesting that restoring autophagic flux may represent a promising therapeutic strategy.

Exosomes, extracellular vesicles carrying diverse cargo (e.g., DNA, non-coding RNAs, lipids), play pivotal roles in disease diagnosis and therapy [10]. Shared molecular machinery between exosome biogenesis and autophagy suggests coordinated regulation of these processes [11]. We hypothesize that exosomes modulate interstitial pulmonary fibrosis through autophagy pathways. Exosomes deliver functional molecules (e.g., miRNAs) to recipient cells via membrane fusion, thereby reprogramming cellular behavior [12,13].

miRNAs, small non-coding RNAs that post-transcriptionally silence target mRNAs by binding their 3′-untranslated regions (3′-UTRs) [14], regulate critical processes such as proliferation, migration, and inflammation [[15], [16], [17], [18]]. While miRNAs are implicated in fibrosis and autophagy [19,20], the role of exosomal miRNAs in IPF remains underexplored.

Core fucosylation (CF), a post-translational glycan modification, governs protein-protein interactions and signaling pathway activation [21,22]. Though CF is well-studied in cancer [23,24], our group first linked CF to IPF pathogenesis via autophagy [9]and senescence [25]. CF modifies fibrotic receptors, including IGF1R, TGFβR, and EGFR [26,27], with IGF1R serving as a key node in the PI3K/AKT autophagy pathway [28].

This study reveals that plasma exosomes from IPF patients exacerbate fibrosis by suppressing AEC autophagy. High-throughput sequencing identified miR-15a-5p as the most significantly downregulated miRNA in IPF exosomes. Bioinformatic analysis and dual-luciferase reporter assays confirmed IGF1R—a CF-modified membrane protein—as a direct miR-15a-5p target. Mechanistically, miR-15a-5p deficiency stabilizes CF-modified IGF1R, activating the PI3K/AKT pathway to inhibit autophagy and drive fibrotic remodeling.

## 2 Materials and methods

### 2.1 Blood collection

Blood was collected from patients with IPF and healthy controls at the First Hospital of Dalian Medical University, which was approved by the Ethics Committee of the First Hospital of Dalian Medical University (Approval No. PJ-KS-KY-2024-342), and informed consent was obtained from the study participants. The basic information of the patients is shown in Table 1 of the Supplementary Information.

### 2.2 Exosome extraction and characterization

Exosomes were isolated via ultracentrifugation, resuspended in PBS, and analyzed by TEM (Hitachi), nanoparticle tracking (NanoFCM), and Western blot (CD9/CD81).

The samples were rapidly re-solubilized at 37 °C and transferred into clean centrifuge tubes, and sequentially subjected to initial centrifugation at 2,000 *g* (4 °C, 30 min) to remove cellular debris, and the supernatant was collected and subjected to secondary centrifugation at 10,000 *g* (4 °C, 45 min) to remove large particulate matter. After filtration through a 0.45 μm pore size filter membrane, the filtrate was enriched for exosomes by ultracentrifugation at 100,000 *g* (4 °C, 70 min), and the precipitate was washed with pre-cooled PBS (10 mL) and then purified by repeated ultracentrifugation. The final precipitate was reconstituted with 200 μL of PBS and partitioned for transmission electron microscopy (20 μL), particle size assay (10 μL), protein extraction (10 μL), and −80 °C freezing.

TEM: 10 μL of exosome suspension was loaded onto a carbon-coated copper mesh, and the excess liquid was removed by filter paper after 60 s of standing. 10 μL of 2 % uranyl acetate solution was used for negative staining, and the floating liquid was removed again after 60 s of standing. The samples were dried by natural evaporation at room temperature, and then imaged by transmission electron microscopy under an accelerating voltage of 100 kV to obtain nanoscale vesicle morphology characteristics.

Nanoparticle tracking:10 μL of exosome suspension was diluted to 30 μL with PBS, and standard particles were used to calibrate the instrument parameters before detection. The sample was diluted according to the gradient and then injected into the sample for detection to avoid clogging of the microfluidic system. The detection system automatically outputs particle size distribution spectra and quantitative data of particle concentration.

### 2.3 Cell culture

MLE_12_ cells (ATCC) were cultured in DMEM/F12 medium supplemented with 10 % fetal bovine serum and 1 % penicillin-streptomycin, while A549 cells (Saibai Kang, China) were maintained in F-12K medium with 10 % fetal bovine serum and 1 % penicillin-streptomycin. Both cell lines were incubated at 37 °C with 5 % CO_2_.

### 2.4 Bioinformatics analysis

Small RNA sequencing libraries were prepared using TruSeq Small RNA Sample Prep Kits (Illumina, San Diego, USA) and sequenced on the Illumina Hiseq 2000/2500 platform (50 bp single-end reads). The base calling software CASAVA (v1.8) for Illumina sequencing platforms assessed sequencing quality using Sanger-format encoded quality scores, where higher values correspond to lower base-calling error rates. In quality evaluation, Q20 (error probability <1 %) yielded a mean quality score of 98.32 % across all samples, while Q30—representing a more stringent standard with an error rate <0.1 %—demonstrated a mean score of 95.49 % under this criterion. Raw data were analyzed via ACGT101-miR (v4.2) (LC Sciences, Houston, Texas, USA), filtered against mRNA, RFam (v12.2), and Repbase (v22.07) databases, and aligned with miRBase for miRNA identification. Differentially expressed miRNAs were predicted using TargetScan (v5.0) [29] and miRanda (v3.3a) [30] and their targets were analyzed using Gene Ontology and KEGG.

### 2.5 Construction of a mouse model of pulmonary fibrosis

CKO mice (FUT8^flox/flox^;Sftpc^CRE^) and Fl/Fl (FUT8^flox/flox^) controls (BIOSETO Ltd., Beijing) were randomly divided into control and bleomycin-induced fibrosis groups (n = 6 each). Lung fibrosis was induced via intratracheal bleomycin (5 mg/kg, Meilun) in BLM groups, while controls received saline. Mice were euthanized after 21 days.

### 2.6 Exocytosis endocytosis assay

Cells were seeded according to the experimental group and cultured until they adhered to the surface. 2 μM PKH67 dye was used to label 10 μg/mL of exosomes. After adding the dye working solution, the centrifuge tube was tightly sealed, mixed for 1 min using a vortex mixer, and then incubated for 10 min. 10 mL of PBS was added and mixed, and the exosomes were extracted again according to the exosome extraction method to remove excess dye. The precipitate is the stained exosome. Cells were treated with exosome labeled with 10 μg/mL PKH67 and incubated at 37 °C and 5 % CO_2_ for 0 h, 1 h, 4 h, 12 h, and 24 h. After washing three times with PBS, images were captured using a fluorescence microscope.

### 2.7 Dual luciferase reporter gene assay

293T cells were seeded in 12-well plates at 1 × 10^5^ cells/well. After 24 h culture, transfection was performed using Lipofectamine™ 3000. The IGF1R-WT or IGF1R-MUT reporter plasmid (1.25 μL) was co-transfected with either mimic NC or miR-15a-5p mimic (37.5 pmol). Complexes were added dropwise to wells followed by crosswise agitation. Cells were maintained at 37 °C/5 % CO_2_ for 48 h. Luciferase activity was quantified using the Dual-Luciferase Reporter Kit (KGI Bio).

### 2.8 Western blotting

Protein concentrations were determined using a BCA assay kit (Beyotime Biotechnology). Equal amounts of protein were separated by 10 % SDS-PAGE and subsequently transferred onto PVDF membranes (Bio-Rad). The membranes were blocked with 4 % skim milk in TBST (10 mM Tris-HCl, 150 mM NaCl, 0.1 % Tween® 20, pH 7.6) at room temperature for 1–2 h. Following blocking, the membranes were incubated overnight at 4 °C with primary antibodies diluted 1:1000 in TBST. After extensive washing, the membranes were incubated with horseradish peroxidase (HRP)-conjugated secondary antibodies diluted 1:1000 in TBST at 37 °C for 2 h. Following additional thorough washes, protein bands were visualized using enhanced chemiluminescence (ECL). Detailed antibody information is provided in the Supplementary Information.

### 2.9 Real-time quantitative polymerase chain reaction (qRT-PCR)

Total RNA was extracted from lung tissues/cells using Trizol reagent. miRNA cDNA was synthesized via tailing. qRT-PCR (Roche LightCycler96, 20 μL SYBR-Green system) quantified gene expression using the 2−ΔΔCT method normalized to U6 or β-actin. Primer sequences are provided in Supplementary Table 2 and transfection sequences in Supplementary Table 3.

### 2.10 Immunofluorescence

Lung tissues were fixed, paraffin-embedded, sectioned, and deparaffinized. Antigen retrieval (10 mM, pH 6.0, citrate buffer/microwave, 15 min) was followed by blocking (FastBlock), primary antibody incubation (4 °C overnight), secondary antibodies (Alexa Fluor 488/555), DAPI staining, glycerol mounting, and fluorescence microscopy imaging.

### 2.11 Immunoprecipitation

Protein samples solubilized in 500 μg specialized lysis buffer (RIPA buffer supplemented with PMSF at a 100:1 ratio) were incubated with either the specific anti-IGF1R antibody or control normal rabbit IgG under optimized conditions for 4–8 h to facilitate antigen-antibody binding. Subsequently, 20 μL of Protein A/G plus-Agarose beads were added to the reaction mixture, followed by continuous rotation overnight at 4 °C. The following day, immunoprecipitated complexes were washed extensively with cold washing buffer (25 mM Tris, 150 mM NaCl, 0.1 % NP-40). During washing steps performed at 4 °C, beads were separated using a magnetic rack and supernatants were discarded. Finally, equal aliquots of proteins from each precipitated sample were subjected to Western blot analysis to evaluate the immunoprecipitation efficiency and specificity of the target protein IGF1R.

### 2.12 Statistical analysis

Data analysis was performed using the professional statistical software GraphPad Prism 10.0. Differences between two groups were analyzed by two-sample Student's t-tests. Comparisons among multiple groups were conducted using one-way analysis of variance. Quantitative data are presented as the mean ± standard deviation (mean ± SD) to characterize dispersion and central tendency. All experiments included three biological replicates. Statistical significance was defined as a probability threshold of p < 0.05.

## 3 Results

### 3.1 Plasma Exosomes Suppress Autophagy and Promote Fibrosis in A549 cells

**Fig. 1:**
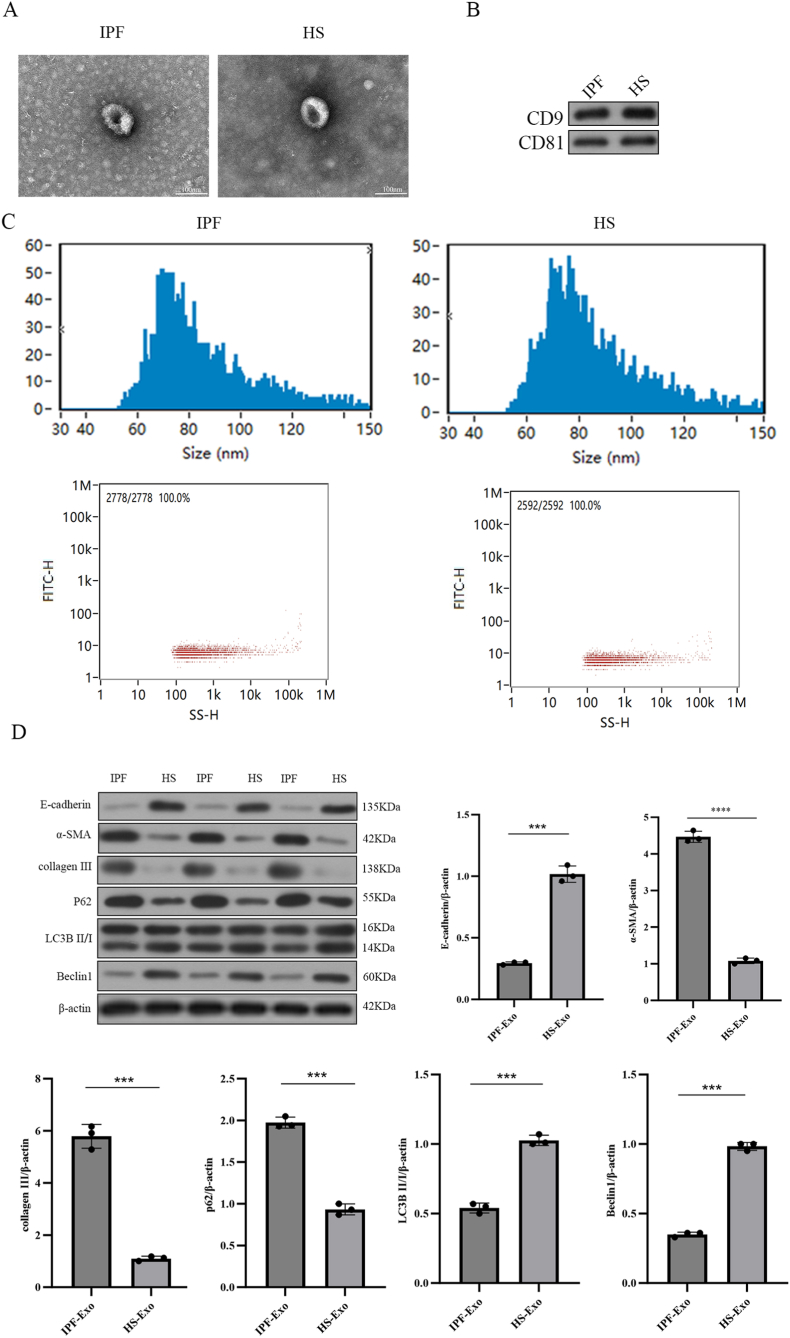
Plasma Exosomes Suppress Autophagy and Promote Fibrosis in A549 Cells. (A) Exosomes were isolated from IPF patient and control plasma via ultracentrifugation. (B) Exosomal markers CD9 and CD81 were confirmed by Western blot. (C) Exosome morphology was visualized by TEM (scale bar: 100 nm). (D) Western blot quantified E-cadherin, α-SMA, Collagen III, p62, LC3B, and Beclin1 levels in cells (β-actin-normalized). Data: mean ± SD; ∗P < 0.05, ∗∗P < 0.01.

Plasma exosomes were isolated from five idiopathic pulmonary fibrosis (IPF) patients and five healthy subjects (HS) via ultracentrifugation. Characterization using transmission electron microscopy (TEM) revealed cup-shaped, double-membrane vesicles (Fig. 1A), while western blot further validated exosomal identity through enriched CD9 and CD81 expression (Fig. 1B). Nanoparticle tracking analysis (NTA) confirmed comparable exosome sizes (IPF: 84.47 nm; HS: 85.02 nm) and concentrations (IPF: 1.31 × 10^9^/mL; HS: 1.22 × 10^9^/mL) (Fig. 1C).

Exosomes can be transported to recipient cells and function [10], and some studies have suggested a link between autophagy and exosome secretion to maintain cellular homeostasis [31]. To investigate functional effects, A549 alveolar epithelial cells were treated with IPF or HS plasma exosomes. The results showed that the IPF group had reduced levels of E-cadherin protein expression, increased levels of α-SMA and CollagenIII proteins, increased levels of P62 protein, and reduced levels of LC3B and Beclin1 expression compared with the control HS group (Fig. 1D). These findings demonstrate that IPF plasma exosomes suppress autophagic flux and exacerbate fibrotic remodeling in recipient alveolar epithelial cells.

### 3.2 Uptake and Functional Role of Plasma Exosomal miR-15a-5p in A549 alveolar epithelial cells

**Fig. 2:**
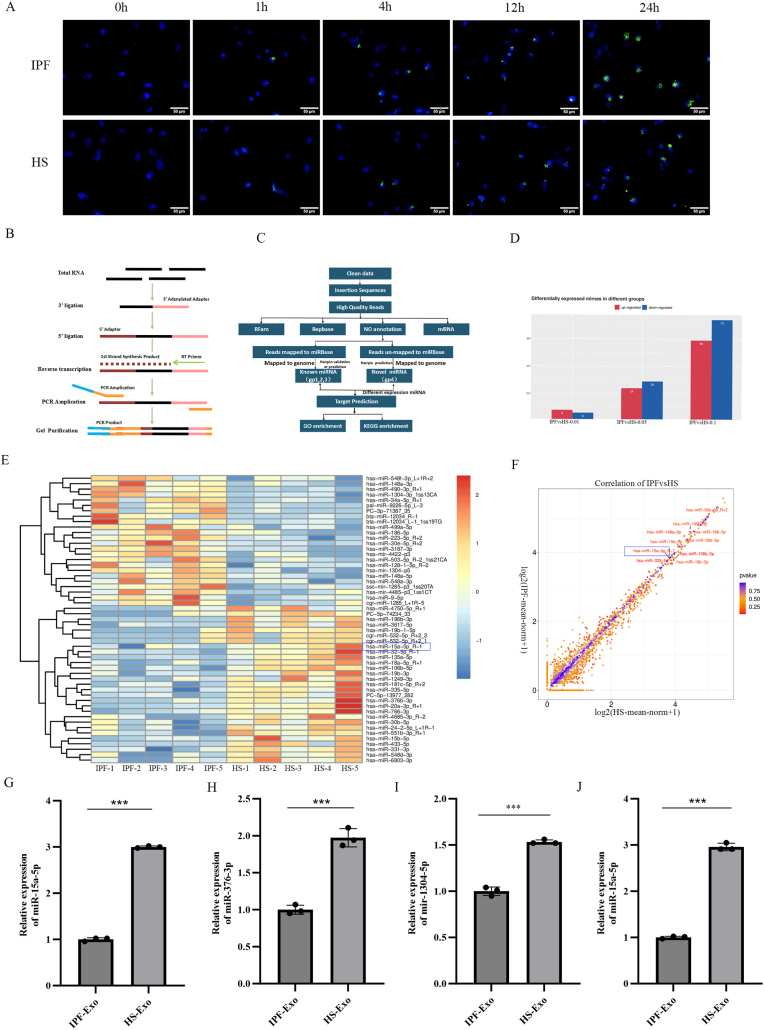
Uptake and Functional Role of Plasma Exosomal miR-15a-5p in A549 Alveolar Epithelial Cells. (A) Exosome uptake in A549 cells was visualized by fluorescence microscopy (scale bar: 50 μm). (B) Schematic of miRNA sequencing workflow. (C) Bioinformatics pipeline for miRNA data analysis. (D) Differentially expressed miRNAs (red: upregulated, blue: downregulated; numbers indicate counts). (E) Heatmap of miRNA expression profiles. Visually display the overall expression levels of all filtered differentially expressed miRNAs across all samples. X-axis: represents IPF samples and HS control samples. Y-axis: represents the filtered differentially expressed miRNAs. (F) Volcano plot highlighting miR-15a-5p as a key downregulated miRNA. (G–I) qRT-PCR confirmed reduced miR-15a-5p and miR-376b-3p, but not mir-1304-5p, in IPF exosomes (vs. HS). (J) IPF exosomes delivered less miR-15a-5p to A549 cells than HS exosomes.

To investigate the cellular internalization of plasma-derived exosomes and their functional impact, PKH67-labeled exosomes were co-cultured with human alveolar epithelial cells (A549). Fluorescence microscopy revealed robust exosomal uptake by A549 cells at 24 h (Fig. 2A). Differentially expressed miRNAs were identified based on two criteria: fold-change magnitude and statistical significance. For analyses with biological replicates, miRNAs with a P < 0.05 were considered statistically significant. In contrast, for analyses lacking biological replicates, the screening threshold required |log_2_ (fold-change)| > 1 (equivalent to fold-change >2 or <0.5) combined with P < 0.05. Subsequent high-throughput sequencing of plasma exosomal miRNAs from IPF patients and HS identified 48 differentially expressed miRNAs (P < 0.05), including 22 upregulated (e.g., mir-1304-5p) and 26 downregulated species (e.g., miR-15a-5p, miR-376b-3p) (Fig. 2B–E). Volcano plot analysis highlighted miR-15a-5p as a prominently downregulated miRNA in IPF-derived exosomes (Fig. 2F).

To validate the sequencing results, we selected three representative differentially expressed miRNAs (miR-15a-5p, miR-376b-3p, and mir-1304-5p) for qRT-PCR validation, which showed that miR-15a-5p and miR-376b-3p were consistent with the sequencing results, but the difference in miR-15a-5p was more significant (Fig. 2G–I). Complementary analysis of the GEO dataset (GSE32538) further demonstrated decreased expression of miR-15a (the precursor of miR-15a-5p) in IPF lung tissues compared to controls. These findings suggest that miR-15a-5p may mediate exosome-dependent regulatory effects in alveolar epithelial cells.

qRT-PCR quantification of miR-15a-5p in plasma exosomes and exosome-treated A549 cells revealed concordant trends: IPF-derived exosomes delivered significantly less miR-15a-5p to A549 cells than HS-derived exosomes (Fig. 2J). This impaired transfer correlated with reduced autophagy and increased fibrotic activity in A549 cells, supporting the hypothesis that exosomal miR-15a-5p depletion contributes to IPF pathogenesis through dysregulation of epithelial cell autophagy and fibrotic signaling.

### 3.3 Functional Role of miR-15a-5p in Regulating Autophagy and Fibrosis in Alveolar Epithelial Cells

**Fig. 3:**
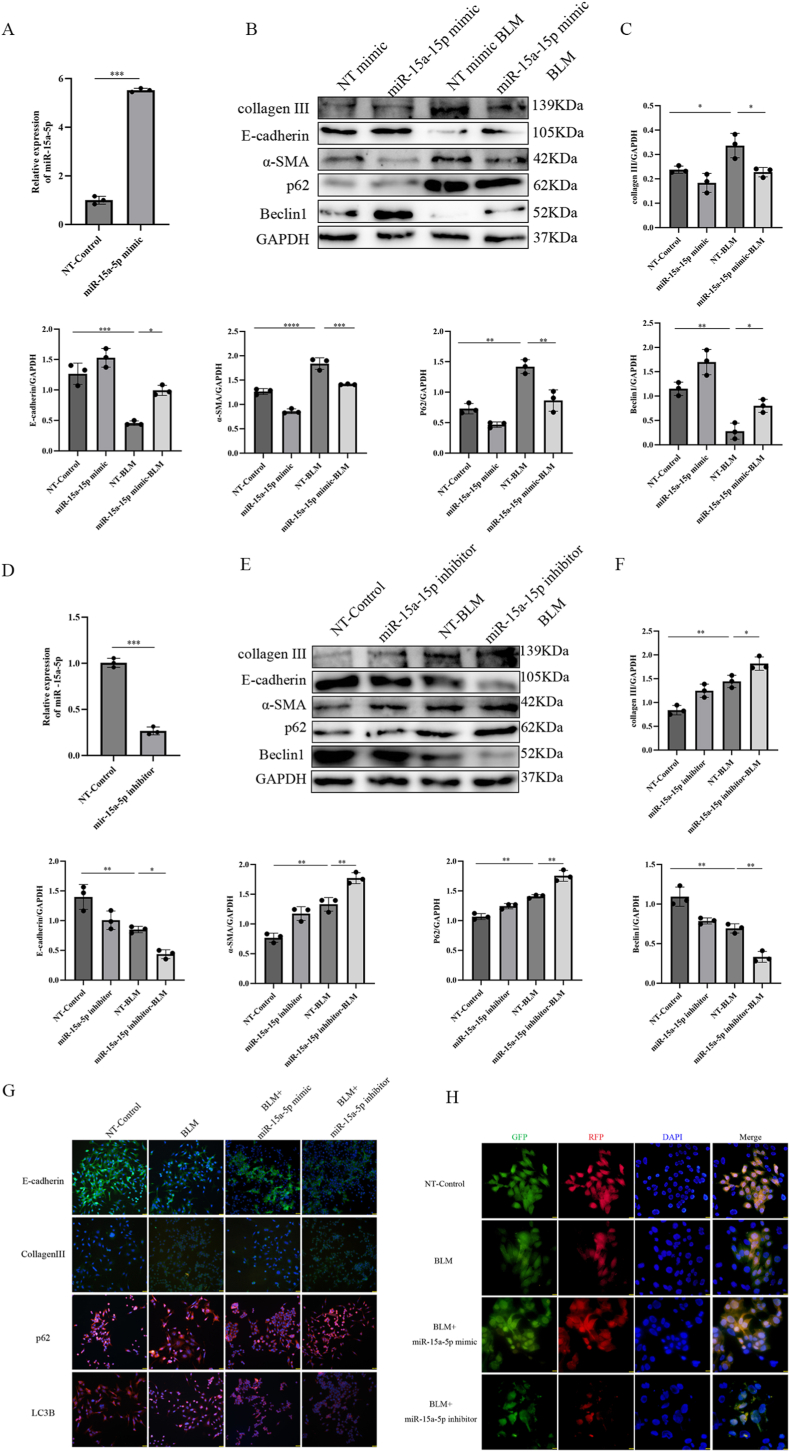
Functional Role of miR-15a-5p in Regulating Autophagy and Fibrosis in Alveolar Epithelial Cells. (A, D) miR-15a-5p transfection efficiency was confirmed by qRT-PCR (∗∗∗P < 0.001). (B–C,E–F) Western blot quantified E-cadherin, α-SMA, Collagen III, p62, and Beclin1 levels (GAPDH-normalized). Data: mean ± SD; ∗P < 0.05, ∗∗P < 0.01, ∗∗∗P < 0.001, ∗∗∗∗P < 0.0001. (G) Immunofluorescence detected E-cadherin, Collagen III, p62, and LC3B expression (scale bar: 50 μm). (H) Fluorescent microscopy was used to observe the dynamic changes in autophagy flux in each group of cells, distinguished by green (GFP), red (RFP), and blue (DAPI) fluorescent labels (scale bar: 25 μm).

To validate the hypothesis that miR-15a-5p modulates autophagy and fibrosis, we employed a BLM-induced epithelial injury model using MLE_12_ cells. MLE_12_ cells were treated with 1 mg/mL BLM to recapitulate fibrotic injury, followed by transfection with miR-15a-5p mimic, inhibitor, or non-targeting (NT) controls. qRT-PCR results showed that compared to the NT control, the expression of miR-15a-5p in the mimic construct was significantly increased (Fig. 3A).

Western blot analysis revealed that BLM-treated MLE_12_ cells exhibited reduced expression of epithelial markers (E-cadherin) and autophagy regulators (Beclin-1), alongside elevated levels of fibrotic markers (α-SMA, Collagen III) and autophagy substrate p62 (Fig. 3B and C). Transfection with miR-15a-5p mimic significantly reversed these BLM-induced changes, restoring E-cadherin and Beclin-1 expression while suppressing α-SMA, Collagen III, and p61. Conversely, qRT-PCR results showed that compared to the NT control, the expression of miR-15a-5p in the inhibitor construct was significantly decreased (Fig. 3D). miR-15a-5p inhibitor exacerbated BLM-driven fibrotic signaling and autophagy impairment (Fig. 3E and F).

Immunofluorescence staining aligned with protein expression trends: BLM diminished E-cadherin and LC3B fluorescence while intensifying Collagen III and p62 signals (Fig. 3G). miR-15a-5p mimic attenuated these effects, whereas the inhibitor amplified BLM-induced epithelial damage and autophagic suppression. To assess autophagic flux, MLE_12_ cells transfected with stubRFP-sensGFP-LC3 lentivirus were analyzed by fluorescence microscopy. BLM treatment reduced both yellow and red puncta, indicating stalled autophagic flux (Fig. 3H). miR-15a-5p mimic restored puncta formation, while the inhibitor further suppressed flux, corroborating its regulatory role in autophagy. The above results suggest that up-regulation of miR-15a-5p improves the inhibitory effect of BLM on autophagy in the BLM-induced alveolar epithelial cell injury model in mice, thereby ameliorating the degree of fibrosis.

### 3.4 Identification of IGF1R as a direct target of miR-15a-5p and Its Post-Translational Regulation by core fucosylation

**Fig. 4:**
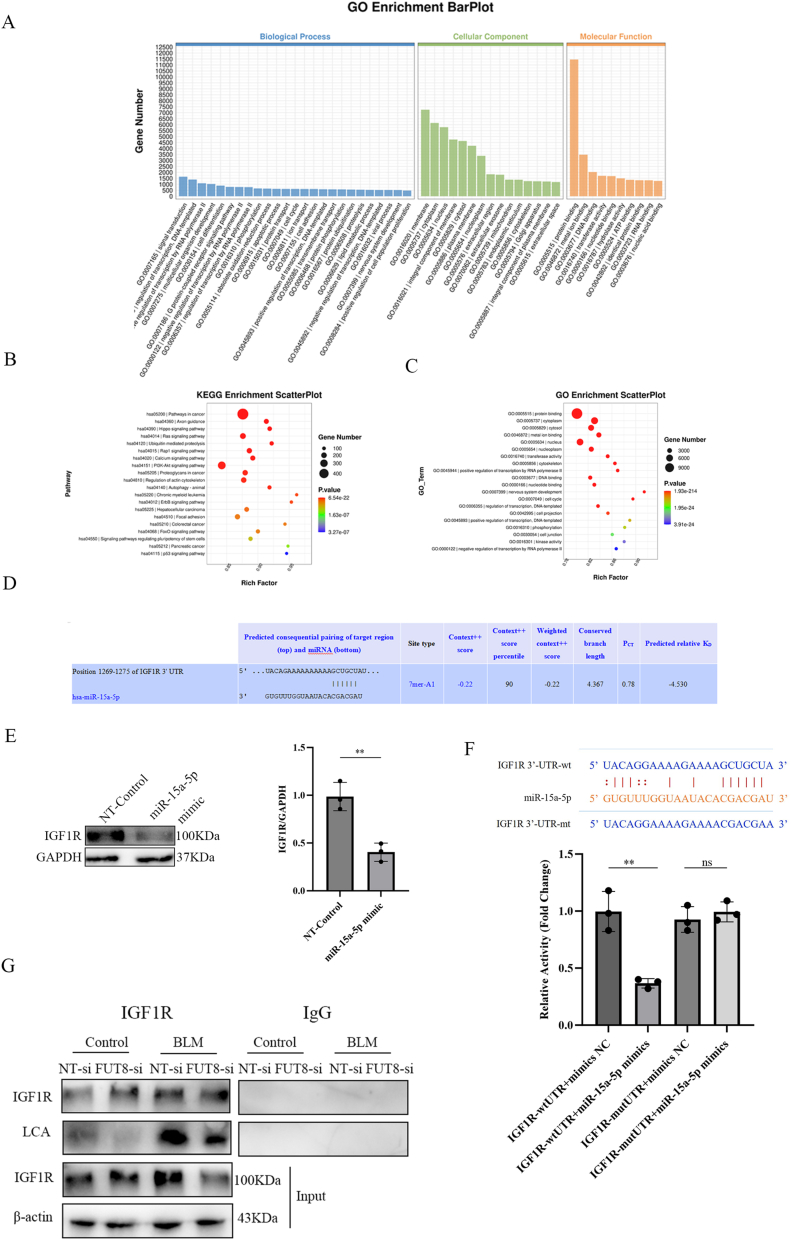
Identification of IGF1R as a Direct Target of miR-15a-5p and Its Post-Translational Regulation by Core Fucosylation. (A) GO enrichment analysis categorized miRNA target genes into molecular function, biological process, and cellular component. (B) KEGG pathway enrichment displayed via bubble plot. (C) GO term enrichment visualized by bubble plot. The horizontal axis represents the Rich factor, which indicates the proportion of target genes located in the GO term relative to the total number of genes in that GO term. A higher Rich factor indicates a higher degree of GO enrichment. The vertical axis represents the GO Term, i.e., the GO functional annotation. In the bubble chart, the size of the bubbles represents the number of S genes, while the color of the bubbles represents the p-value of the enrichment analysis, i.e., the significance of the enrichment. A smaller p-value indicates a more significant enrichment. (D) TargetScan predicted miR-15a-5p binding sequence in IGF1R 3′UTR. (E) IGF1R protein levels were quantified by Western blot (mean ± SD; ∗P < 0.05, ∗∗P < 0.01). (F) Dual-luciferase assay confirmed miR-15a-5p binding to IGF1R 3′UTR. (G) IGF1R-specific antibodies were used for immunoprecipitation.

To elucidate the molecular mechanism underlying miR-15a-5p′s role in autophagy regulation, we performed bioinformatic screening using TargetScan (v5.0) and miRanda algorithms. Intersection analysis identified IGF1R—a key modulator of autophagy signaling—as a high-confidence target (Fig. 4A–C). Computational prediction revealed a conserved miR-15a-5p binding site within the IGF1R 3′-untranslated region (UTR) (Fig. 4D).

Experimental validation via western blot confirmed that miR-15a-5p mimic transfection significantly suppressed IGF1R protein expression in MLE_12_ cells compared to negative controls (NC) (Fig. 4E). To establish direct targeting, dual luciferase reporter assays were conducted by cloning wild-type (IGF1R-wtUTR) or mutant (IGF1R-mutUTR) 3′UTR sequences into psiCHECK2 vectors. Co-transfection of miR-15a-5p mimic with IGF1R-wtUTR reduced luciferase activity versus mimic NC, whereas no significant change occurred with IGF1R-mutUTR (Fig. 4F), confirming sequence-specific repression.

CF, a critical post-translational modification of proteins, has been shown to mediate targeted effects in IPF through autophagy regulation. To investigate whether CF modulates autophagy pathways via IGF1R modification, we performed lectin pulldown assays using Lens culinaris agglutinin (LCA), which specifically recognizes α1-6 fucosylated N-glycans [32]. Result revealed significantly elevated LCA-binding signals in BLM-treated MLE_12_ cells compared to non-targeting siRNA controls (Fig. 4G), indicating enhanced α1-6 fucosylation on IGF1R. Notably, siRNA-mediated knockdown of FUT8 (encoding α1-6 fucosyltransferase, the sole enzyme responsible for CF) effectively attenuated BLM-induced LCA signal enhancement in MLE_12_ cells (Fig. 4G). These findings demonstrate that IGF1R undergoes CF modification in alveolar epithelial cells.

### 3.5 CF modulates BLM-Induced Pulmonary Fibrosis via IGF1R-Dependent Autophagy Regulation

**Fig. 5:**
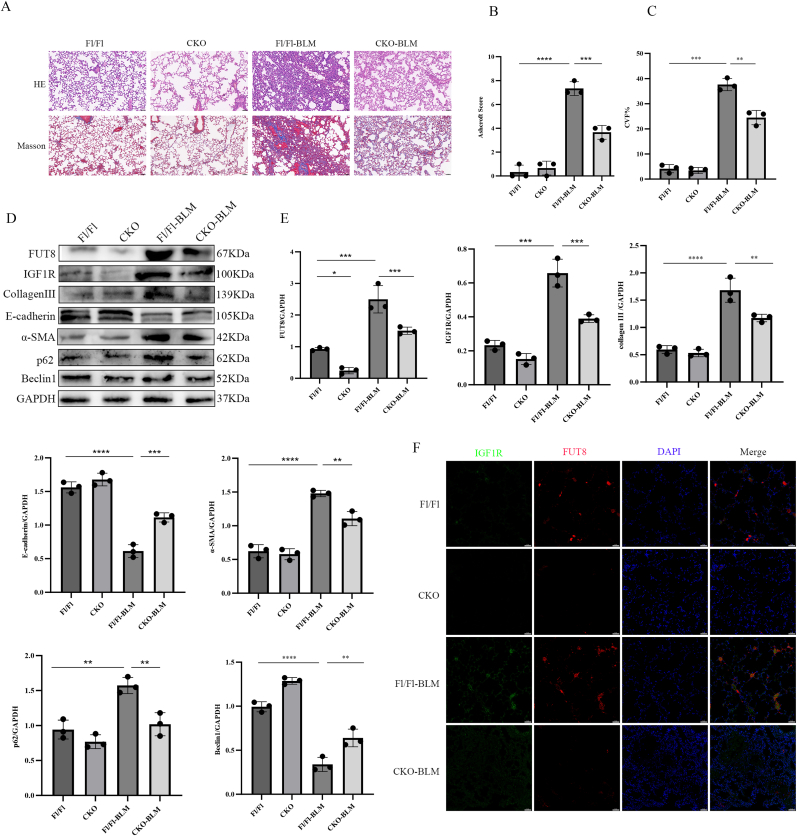
CF Modulates BLM-Induced Pulmonary Fibrosis via IGF1R-Dependent Autophagy Regulation. (A) Mouse lung tissue sections underwent HE and Masson staining (scale bar: 50 μm; data: mean ± SD; ∗∗∗P < 0.001, ∗∗∗∗P < 0.0001). (B) Fibrosis severity was scored via Ashcroft criteria. (C) Collagen volume fraction (CVF%) was calculated from Masson staining. (D–E) FUT8, IGF1R, E-cadherin, α-SMA, Collagen III, p62, and Beclin1 protein levels were analyzed by Western blot (GAPDH-normalized). (E) FUT8 mRNA levels in Fl/Fl vs. CKO mice were assessed by qRT-PCR. (F) IGF1R and FUT8 co-localization in lung tissues was confirmed by immunofluorescence (scale bar: 50 μm).

To investigate the functional impact of CF on IGF1R in pulmonary fibrosis, we employed a BLM-induced murine model using conditional FUT8 knockout (CKO) mice. Mice were anesthetized with intraperitoneal pentobarbital sodium (60 mg/kg), followed by intratracheal instillation of BLM (5 mg/kg) to induce alveolar epithelial injury. Histopathological analysis at day 21 revealed attenuated alveolar architecture disruption and reduced collagen deposition in BLM-treated CKO mice compared to BLM-Fl/Fl, as evidenced by hematoxylin-eosin (HE) and Masson's trichrome staining (Fig. 5A). Quantitative assessments confirmed these findings: Ashcroft scoring of HE sections demonstrated milder fibrosis in CKO mice (Fig. 5B), while collagen volume fraction (CVF%) analysis of Masson-stained sections showed significantly less extracellular matrix accumulation (Fig. 5C).

Western blotting of lung tissues demonstrated elevated IGF1R, α-SMA, Collagen III, and p62 protein levels alongside reduced E-cadherin and Beclin1 expression in BLM-treated Fl/Fl mice versus saline controls. Strikingly, FUT8 ablation in CKO mice reversed these BLM-induced changes, restoring E-cadherin and Beclin1 while suppressing IGF1R, α-SMA, and p62 (Fig. 5D and E). Immunofluorescence co-staining further revealed enhanced IGF1R (green) and FUT8 (red) fluorescence in BLM-exposed Fl/Fl lungs, suggesting BLM-driven upregulation of both proteins. This co-localization was abolished in CKO mice, with IGF1R and FUT8 signals returning to baseline levels (Fig. 5F).

These data establish that CF-mediated post-translational modification of IGF1R critically regulates autophagy flux in BLM-induced fibrosis. FUT8 ablation mitigates fibrotic progression by restoring IGF1R-dependent autophagic activity, positioning CF-IGF1R axis disruption as a novel therapeutic strategy for IPF.

### 3.6 CF regulates BLM-induced alveolar epithelial injury via IGF1R-Dependent Autophagy Modulation

**Fig. 6:**
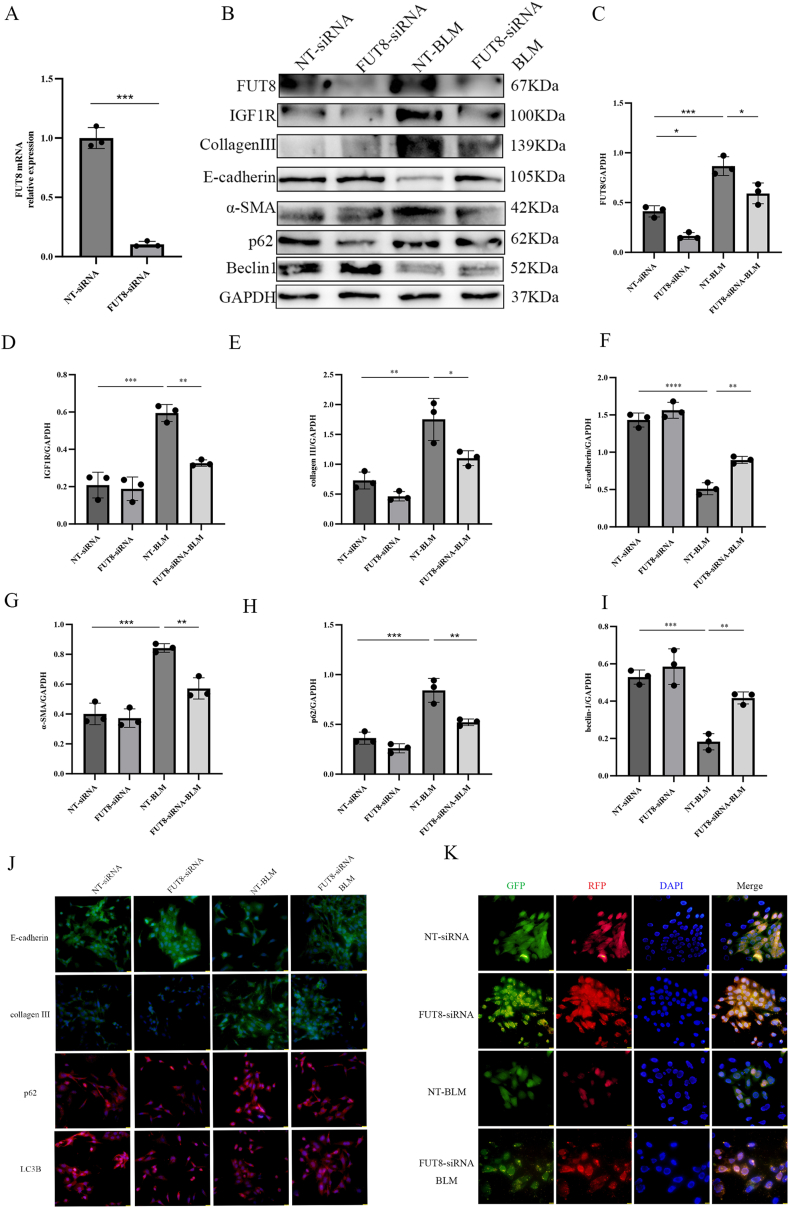
CF Regulates BLM-Induced Alveolar Epithelial Injury via IGF1R-Dependent Autophagy Modulation. (A) si-FUT8 transfection efficiency was validated by qRT-PCR. (B–I) FUT8, IGF1R, E-cadherin, α-SMA, Collagen III, p62, and Beclin1 protein levels were quantified by Western blot (GAPDH-normalized). (J) Immunofluorescence detected E-cadherin, Collagen III, p62, and LC3B expression (scale bar: 50 μm). (K) Autophagic flux dynamics were observed via fluorescence microscopy (scale bar: 25 μm).

To validate whether CF impacts autophagy and fibrosis through IGF1R modification, we established a BLM-induced alveolar epithelial injury model using MLE_12_ cells. Cells were transfected with non-targeting siRNA (NT siRNA) or FUT8 siRNA, followed by BLM treatment. qRT-PCR confirmed >70 % knockdown of FUT8 siRNA-treated cells, indicating successful siRNA transfection and target gene silencing (Fig. 6A).

Western blot analysis revealed that BLM-exposed NT siRNA-transfected cells exhibited elevated expression of fibrotic markers (α-SMA, Collagen III), p62, and IGF1R, alongside reduced E-cadherin and Beclin1 markers (Fig. 6B–I). Strikingly, FUT8 siRNA transfection reversed these BLM-induced changes, suppressing α-SMA, Collagen III, p62, and IGF1R while restoring E-cadherin and Beclin1 expression (Fig. 6A–H). Immunofluorescence staining corroborated these findings: BLM-treated NT siRNA-transfected cells showed intensified Collagen III and p62 signals with attenuated E-cadherin and LC3B fluorescence, whereas FUT8 siRNA mitigated these effects (Fig. 6J).

To delineate the stage of autophagy inhibition, MLE_12_ cells transfected with stubRFP-sensGFP-LC3 lentivirus were analyzed by fluorescence microscopy. BLM treatment reduced both yellow and red puncta, indicative of impaired autophagic flux (Fig. 6K). FUT8 siRNA partially rescued this defect, restoring puncta formation to near-baseline levels (Fig. 6K).

Collectively, these data establish that CF-mediated IGF1R modification drives BLM-induced epithelial injury by suppressing autophagic flux. FUT8 ablation alleviates fibrosis by restoring IGF1R-dependent autophagy activity, mirroring in vivo observations in murine lung tissues.

### 3.7 miR-15a-5p Downregulation Exacerbates Pulmonary Fibrosis via CF-Modulated IGF1R/PI3K/AKT Axis-Dependent Autophagy Suppression

Our earlier findings demonstrated that miR-15a-5p regulates pulmonary fibrosis by targeting IGF1R-mediated autophagy, while IGF1R itself undergoes CF, a post-translational modification that exacerbates fibrotic progression. We therefore hypothesized that CF acts as an intermediary mechanism linking miR-15a-5p to IGF1R signaling. To test this, MLE_12_ cells were transfected with miR-15a-5p inhibitor or negative control (NC), followed by co-transfection with FUT8 siRNA or NC.

**Fig. 7:**
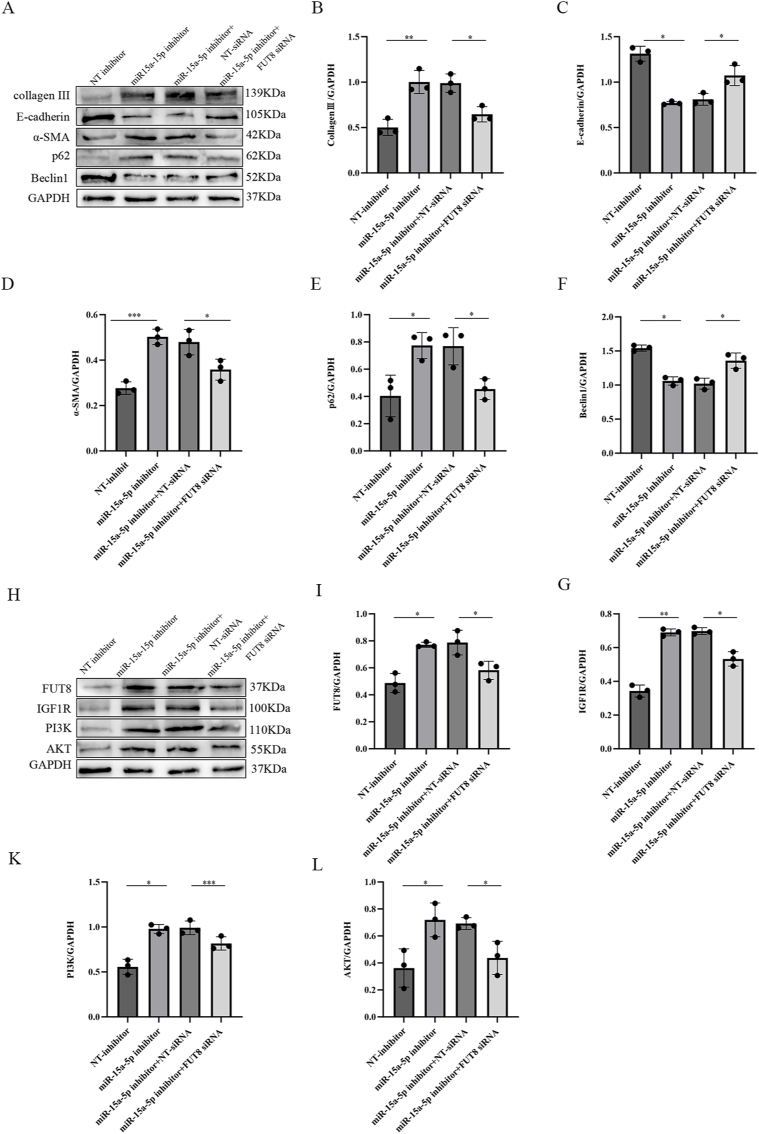
miR-15a-5p Downregulation Exacerbates Pulmonary Fibrosis via CF-Modulated IGF1R/PI3K/AKT Axis-Dependent Autophagy Suppression. (A, H) Protein expression levels of E-cadherin, α-SMA, Collagen III, P62, Beclin1, FUT8, IGF1R, PI3K, and AKT were analyzed and quantified by immunoblotting in each group of cells. (B–F, I–L) Protein expression levels in each group of cells were analyzed semi-quantitatively by protein immunoblotting and normalized using GAPDH as an internal reference. Data were expressed as mean ± standard deviation, ∗ represents P < 0.05, ∗∗ represents P < 0.01. ∗∗∗ represents P < 0.001, ∗∗∗∗ represents P < 0.0001.

Western blot analysis revealed that miR-15a-5p inhibition upregulated fibrotic markers (α-SMA, Collagen III, p62) and downregulated epithelial (E-cadherin) and autophagic (Beclin1) proteins. Notably, FUT8 siRNA co-transfection reversed these effects (Fig. 7A–F). Further profiling showed that miR-15a-5p inhibition elevated FUT8, IGF1R, PI3K, and AKT levels, all of which were normalized by FUT8 knockdown (Fig. 7H–L).

These data establish a hierarchical regulatory axis: miR-15a-5p downregulation promotes CF-dependent IGF1R stabilization, which activates the PI3K/AKT pathway to suppress autophagy, thereby driving alveolar epithelial injury and fibrotic remodeling. FUT8 ablation disrupts this cascade, restoring autophagic flux and attenuating fibrosis.

## 4 Discussion

Pulmonary fibrosis, a common end-stage pathology of numerous lung diseases, remains poorly understood mechanistically. This condition is associated with dismal prognosis and high mortality [33]. Accumulating evidence highlights insufficient alveolar epithelial cell (AEC) autophagy as a pivotal driver of fibrotic progression [34]. Exosomes, owing to their unique biological functions, play critical roles in disease diagnosis and therapy [10]. Shared molecular machinery between exosome biogenesis and autophagy suggests coordinated regulation of these processes [11]. While exosome-mediated autophagy has been studied in other fibrotic tissues [35,36], its role in pulmonary fibrosis warrants deeper exploration. Exosomes regulate interstitial fibrosis by delivering miRNAs to recipient cells, with exosomal miRNAs exhibiting superior stability compared to circulating miRNAs [37]. This discovery not only expands our understanding of miRNA-mediated intercellular communication but also identifies novel molecular targets for antifibrotic therapies [38].

To elucidate the mechanism by which plasma exosomes modulate AEC autophagy in pulmonary fibrosis, we identified differentially expressed miRNAs in IPF patients versus HS via high-throughput sequencing. Among candidates (miR-15a-5p, miR-1304-5p, miR-376b-3p), miR-15a-5p showed the most significant downregulation, validated by qRT-PCR. Although miR-15a-5p has been implicated in other fibrotic diseases [39,40], its role in IPF was previously undefined. Subsequent experiments using PKH67-labeled exosomes confirmed that exosomal miR-15a-5p delivery directly modulates AEC function. Silencing or overexpressing miR-15a-5p in murine AECs demonstrated that miR-15a-5p deficiency suppresses autophagy and exacerbates fibrosis, as evidenced by Western blotting and immunofluorescence.

Mechanistically, bioinformatic screening (KEGG/GO analysis) identified IGF1R—a key receptor in the PI3K/AKT/mTOR autophagy pathway [41]—as a miR-15a-5p target. Dual-luciferase assays confirmed direct binding of miR-15a-5p to the IGF1R 3′-UTR, and no conserved binding sites were identified in the 3′UTR of FUT8, PIK3CA, PIK3R1, and AKT1/2/3. This confirms that miR-15a-5p primarily exerts its regulatory function by directly targeting IGF1R. Our prior work established that core fucosylation (CF) regulates AEC autophagy and fibrosis [9]. Here, co-immunoprecipitation and fluorescence co-staining revealed CF modification of IGF1R in lung tissues and AECs. Using FUT8-knockout (CKO) mice and si-FUT8-transfected AECs, we demonstrated that CF ablation attenuates bleomycin (BLM)-induced fibrosis by suppressing IGF1R. Rescue experiments further showed that miR-15a-5p downregulation promotes CF-dependent IGF1R stabilization, activating PI3K/AKT signaling to inhibit autophagy and exacerbate fibrosis. Current data, validated through multidimensional analysis, indicate that the core target of miR-15a-5p is IGF1R; CF mutation can influence the PI3K/AKT pathway mediated by IGF1R; and cascade disruption (CKO mice) can terminate the fibrotic process. We have made every effort to exclude indirect effects through existing experimental strategies; however, the sequence of molecular events requires further refinement, and we will continue to refine the details of molecular interactions in the future.

This study utilized multi-dimensional evidence, including exosome tracing experiments, the alveolar epithelial cell line (A549/MLE_12_) model, conditional gene knockout mice, and a specific reporting system, to confirm that plasma exosomes can be targeted for delivery to alveolar epithelial cells and regulate autophagy and fibrosis processes through the miR-15a-5p they carry. This indicates that this molecule exhibits significant specificity toward alveolar epithelial cells in the pulmonary environment. Although exosome-mediated intercellular communication is widespread—other cells or tissues may also undergo similar uptake and downstream effects—the surface receptor profile, differentiation state, and tissue microenvironment of the recipient cells decisively influence their interpretation of the same exosome signal [42]. For example, macrophages may activate inflammatory pathways [43], while fibroblasts tend to promote fibrotic responses [44], which fundamentally differs from the barrier function regulation of alveolar epithelial cells. It is important to emphasize that this study focuses on elucidating the specific mechanism by which plasma exosome miR-15a-5p binds to the CF-modified IGF1R receptor on alveolar epithelial cells, thereby activating the PI3K/AKT pathway to regulate autophagy and fibrosis. This pathway may exhibit tissue specificity due to differences in cell type and microenvironment, and its universality requires validation in future studies involving epithelial cells or stem cells in organs such as the kidneys and liver.

This study integrates complementary disease models to elucidate the role of CF modification in IPF: in vitro, BLM-stimulated MLE_12_ cells are used to simulate epithelial damage in the early stages of IPF, focusing on the regulation of alveolar epithelial autophagy and fibrosis by miR-15a-5p and CF modification; in vivo, a 21-day mouse model induced by BLM is used to reproduce the late-stage fibrosis phenotype, to assess the improvement of collagen deposition and lung function by inhibiting CF modification and regulating IGF1R. This dual-model system establishes a “mechanism-phenotype” validation loop: in vitro, precise manipulation eliminates microenvironmental interference to reveal the autonomous function of target molecules; in vivo, it confirms the therapeutic potential of miR-15a-5p in physiological systems. Together, they validate the theoretical framework that “target molecules drive epithelial damage and promote fibrosis”.

This study provides the first evidence of CF modification's role in exosome-mediated IPF pathogenesis, yet certain limitations persist. Although we identified IPF-associated exosomal miRNAs and validated their functional impact on AECs, the cellular origins of plasma exosomes remain undefined. Single-cell RNA sequencing of IPF lung tissues will be employed in future work to trace exosome-producing cells. Additionally, while our preliminary analysis with 10 patient samples achieved statistical significance (P < 0.05), large-scale cohort studies are required to validate these findings and assess their clinical translatability.

## 5 Conclusion

In this study, we demonstrated that plasma exosomal miR-15a-5p is significantly downregulated in IPF patients. Mechanistically, miR-15a-5p deficiency promotes core fucosylation (CF)-dependent stabilization of the membrane protein IGF1R, which activates the PI3K/AKT pathway to suppress alveolar epithelial cell autophagy, thereby exacerbating pulmonary fibrosis. These findings establish miR-15a-5p as a pivotal regulator of CF-mediated IGF1R signaling and autophagic dysregulation in IPF. Collectively, our work identifies miR-15a-5p restoration as a promising therapeutic strategy to attenuate fibrosis by targeting the IGF1R/PI3K/AKT autophagy axis.

## CRediT authorship contribution statement

**Yina Li:** Writing – review & editing, Writing – original draft, Visualization, Software, Methodology, Investigation, Formal analysis, Conceptualization. **Nan Wang:** Writing – original draft, Conceptualization. **Jinying Hu:** Data curation, Conceptualization. **Minlan Luo:** Software, Investigation. **Na Zhang:** Validation, Supervision. **Lili Gao:** Supervision, Resources, Project administration, Funding acquisition, Data curation, Conceptualization.

## Ethics approval and consent to participate

Blood samples were obtained from patients with IPF and healthy controls at the First Affiliated Hospital of Dalian Medical University, which were approved by the Ethics Committee of the First Affiliated Hospital of Dalian Medical University (Approval No. PJ-KS-KY-2024-342), and informed consent was obtained from the study participants.

All animal experiments followed the Guidelines for the Husbandry and Use of Laboratory Animals. The experimental protocol was approved by the Ethics Committee for Animal Experiments of Dalian Medical University (Ethics No. AEE24008).

## Funding

This research was funded by the 10.13039/100018696Dalian Life and Health Sector Mentoring Program project (Project Number: 2024ZDJH01PT063).

## Declaration of competing interest

The authors have no conflicts of interest.

## Figures and Tables

**Table:** 

IPF	Idiopathic pulmonary fibrosis
HS	healthy subjects
miRNA	MicroRNA
CF	Core fucosylation
a-SMA	a-smooth muscle actin
CKO mice	conditional knockout mice
3′-UTR	3′-untranslated regions
qRT-PCR	Real-time quantitative PCR
IGF1R	Insulin-like growth factor 1
PI3K/AKT	phosphoinositide 3-kinase-Akt
BLM	bleomycin
FUT8	fucosyltransferase 8
LCA	Lens Culinaris Agglutinin
MLE_12_	murine lung epithelial-12
A549	Human Non-Small Cell Lung Cancer Cells

## Data Availability

Data will be made available on request.
