# Supplementary material for: The mechanism of plasma exosome miR-15a-5p targeting the CF-modified protein IGF1R to regulate alveolar epithelial autophagy and influence pulmonary interstitial fibrosis
Source: Noncoding RNA Res. 2025 Jul 3;15:51–64. doi: 10.1016/j.ncrna.2025.07.001 (PMC12312048; doi:10.1016/j.ncrna.2025.07.001)
Supplement: Multimedia component 1 [file mmc1.docx]

**supplementary information**

**Blood collection**

Blood was processed within 1 hour after blood collection and centrifuged at 1200×g for 10 min to obtain the upper plasma sample, then the plasma was transferred to 1.5 mL enzyme-free centrifuge tubes and centrifuged at 13,000×g for 2 min, and the separated supernatant was transferred to a specification enzyme-free, sterile, ultra-low-temperature threaded-port resistant cryopreservation tube at -192°C and cryopreserved at -80°C for the subsequent isolation of Exosomes Selection of patients with IPF: A definitive diagnosis of IPF was made by respiratory function testing, HRCT scanning (possible UIP mode), bronchoalveolar lavage (if available), and the patient's clinical history, according to the international recommendations of ATS1.

**Table 1** Plasma exosomes in the IPF and HS groups (age of subjects at time of collection). Lung function (FEV1, FVC, FEV1/FVC%) and diffusion capacity (DLCO) were obtained from the medical records of the subjects closest to the time of sampling.

| Subject number | Age | FEV1 (liters) | FVC  (liters) | FEV1/FVC  (%) | DLCO  (%) |
| --- | --- | --- | --- | --- | --- |
| IPF-1 | 74 | 1.09 | 1.21 | 97.24 | 21.2 |
| IPF-2 | 81 | 2.33 | 2.72 | 85.75 | 47.5 |
| IPF-3 | 68 | 2.38 | 2.94 | 90.35 | 63.8 |
| IPF-4 | 51 | 2.35 | 2.85 | 82.60 | 53.9 |
| IPF-5 | 71 | 2.34 | 2.37 | 89.64 | 61.4 |
| HS-1 | 64 | 3.02 | 3.43 | 84.34 | 81.7 |
| HS-2 | 71 | 3.07 | 3.69 | 83.23 | 91.3 |
| HS-3 | 48 | 2.54 | 2.99 | 84.81 | 89.0 |
| HS-4 | 61 | 3.75 | 4.45 | 85.64 | 96.2 |
| HS-5 | 41 | 2.34 | 2.92 | 85.97 | 81.3 |

**Table 2** Primer sequences

| Gene | sequences |
| --- | --- |
| FUT8 | F 5’-AATACTTGATTCGTCCACAAC-3’  R 5’-CTTCTGTTCCCACTTTGTCTG-3’ |
| β-actin | F 5’-CATCCGTAAAGACCTCTATGCCAAC-3’  R 5’-ATGGAGCCACCGATCCACA-3’ |
| miR-15a-5p | F 5’-TAGCAGCACATAATGGTTTGTG-3’ |
| miR-376b-3p | F 5’-ATCATAGAGGAAAATCCATGTT-3’ |
| mir-1304-5p | F 5’-TTTGAGGCTACAGTGAGATGTG-3’ |

**Table 3** Transfection sequences

| Gene | sequences |
| --- | --- |
| FUT8 siRNA | 5’ GCUACUGAUGAUCCUACUUTT 3’  5’ AAGUAGGAUCAUCAGUAGCTT 3’ |
| miR-15a-5p mimc | 5’ UAGCAGCACAUAAUGGUUUGUG3’  5’ CAAACCAUUAUGUGCUGCUAUU3’ |
| miR-15a-5p inhibitor | 5’ CACAAACCAUUAUGUGCUGCTA3’ |

**Western blotting**

**antibodies :** anti-E-cadherin (ab76055, Abcam, Cambridge, UK), anti-FUT8 (sc-271244, Santa Cruz Biotechnology, Dallas, TX, USA), anti-LCA (B-1045 Vector Laboratories, Berlin Gaim, CA, USA), anti-Collagen III (22734-1-AP, Proteintech, Rosemont, Italy), anti-β-actin (TA-09, ZSGB-Bio, Beijing, China), anti-IGF1R (9750, Cell Signaling Technology, USA), anti-p62 (ab109012, Abcam, Cambridge, UK), anti-Beclin1 (ab207612, Abcam, Cambridge, UK), anti-α-SMA (14395-1-AP, Proteintech, Rosemont, Italy), and anti-GAPDH (10494-1-AP. Proteintech, Rosemont, Italy).

Subsequently, the secondary antibodies corresponding to the primary antibodies - HRP-labeled goat anti-rabbit IgG (ZB-2301, ZSGB-Bio, Beijing, China) or HRP-labeled goat anti-mouse IgG (A0216, Beyotime Biotechnology, Shanghai, China) - - Incubation was performed. Next, signals on the membrane were visualized using an ECL chemiluminescence kit (SW2040, Solarbio, Beijing, China) and image capture was performed by an Analytik Jena VisionWorks system (UVP GelSolo, Analytik Jena AG, Jena, Germany).
